# Delayed Presentation of Traumatic Diaphragmatic Rupture with Herniation of the Left Kidney and Bowel Loops

**DOI:** 10.1155/2013/814632

**Published:** 2013-07-15

**Authors:** Amiya Kumar Dwari, Abhijit Mandal, Sibes Kumar Das, Sudhansu Sarkar

**Affiliations:** ^1^Department of Pulmonary Medicine, Bankura Sammilani Medical College, Bankura, West Bengal 722101, India; ^2^Department of Pulmonary Medicine, Medical College, 88 College Street, Kolkata, West Bengal 700073, India; ^3^Department of Surgery, Bankura Sammilani Medical College, Bankura 722101, India

## Abstract

Rupture of the diaphragm mostly occurs following major trauma. We report a case of delayed presentation of traumatic diaphragmatic hernia on the left side in a 44-year-old male who presented two weeks after a minor blunt trauma. Left kidney and intestinals coils were found to herniate through the diaphragmatic tear. This case demonstrates the importance of considering the diagnosis in all cases of blunt trauma of the trunk. It also illustrates the rare possibility of herniation of kidney through the diaphragmatic tear.

## 1. Introduction

Traumatic diaphragmatic hernias (DH) represents only small percentage of all diaphragmatic hernias but it is no longer an uncommon entity. Injury is mostly caused by severe blunt or penetrating trauma [1]. DH may be recognized during the period of hospitalization immediately following trauma. If the diaphragmatic injury is not recognized during the immediate posttraumatic period, the patient may recover and remain symptom free or present either with chronic thoracoabdominal symptoms or with acute emergency due to intestinal strangulation [2]. During the delayed presentation with chronic thoracoabdominal symptoms, the trauma responsible for the injury is often forgotten and the diagnosis is not suspected. A careful history, physical examination, and awareness of the possibility are the prerequisite for timely diagnosis.

Abdominal organs that commonly herniate are stomach, spleen, liver, mesentery, and small and large bowels. Kidney is rarely found to herniate through the diaphragmatic tear [3]. The case is unique due to occurrence of the DH with minor trauma, its delayed presentation, and herniation of the left kidney into the thorax.

## 2. Case Report

A 44-year-old male patient was kicked in his left lower chest and upper abdomen by a neighbour during a family quarrel. Considering it to be a minor trauma, he continued his daily activities for the next two weeks. He presented to pulmonary medicine outpatient department with left sided dull aching chest pain and nonproductive cough for ten days. There was no history of abdominal pain or haematuria. On examination, he was afebrile but dyspneic (MMRC grade 2) with respiratory rate of 22 breaths/min, oxygen saturation of 96% with room air, pulse rate of 90/min, and blood pressure of 138/84 mm of Hg. On examination of the chest, there was dull note over left infraclavicular area and bowel sounds were audible over the left side of the chest. Examination of other systems was within normal limits.

**Figure 1:**
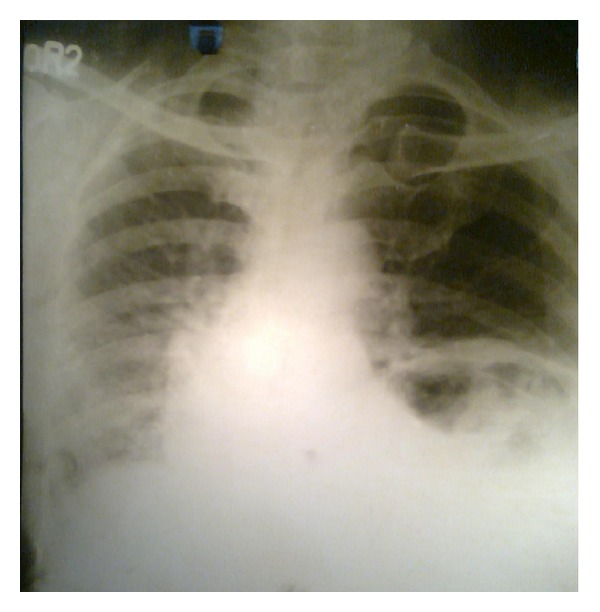
Chest X-ray PA view showing a heterogeneous opacity in the left lower zone.

**Figure 2:**
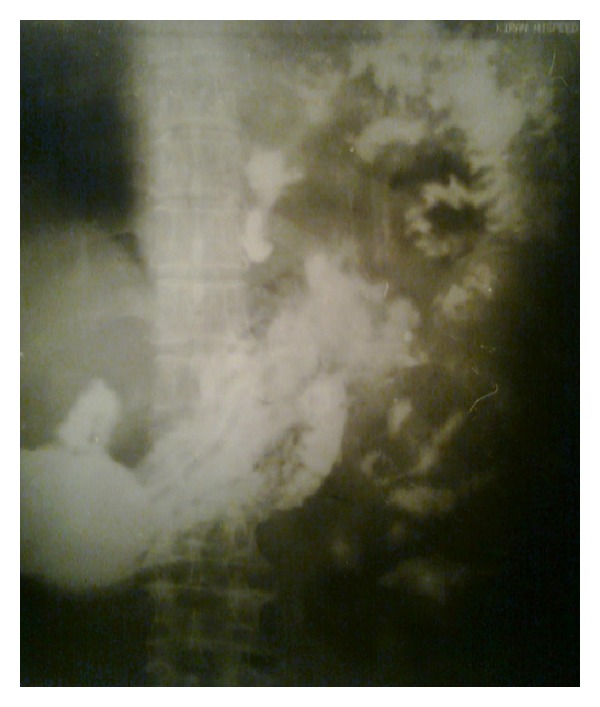
Barium meal examination of stomach and intestine showing presence of loops of intestine within the left hemithorax.

**Figure 3:**
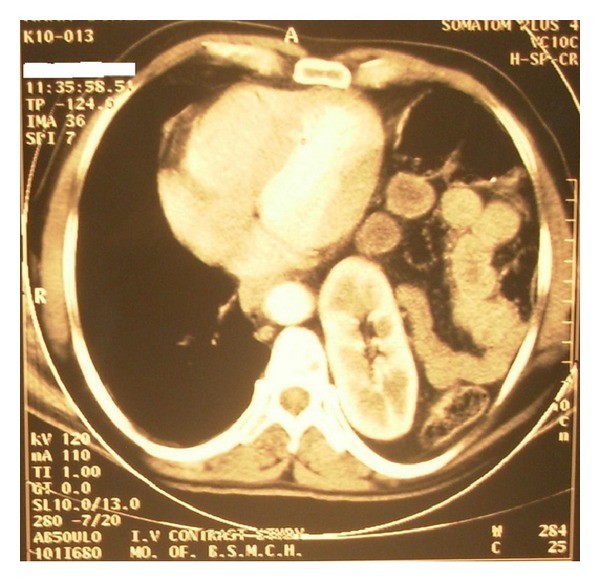
Computed tomography scan of thorax showing presence of bowel loops and kidney in the left hemithorax.

His chest X-ray PA view revealed a heterogeneous opacity in left lower zone but no evidence of any fracture of rib (Figure 1). He was admitted in the chest indoor. Barium meal examination of stomach and intestine revealed presence of loops of intestine within the left hemithorax (Figure 2). USG of abdomen revealed empty left renal fossa and no free fluid in abdomen. Computed tomography scan of thorax showed presence of bowel loops and kidney in the left hemi-thorax (Figure 3). He was diagnosed to have traumatic left diaphragmatic rupture (DR) with herniation of the intestine and left kidney. 

He was shifted to the surgery ward and an emergency thoracoabdominal exploration was performed in the operation theatre. At operation, a rent of 8 cm length with irregular margin was detected in the posterolateral part of the left dome of the diaphragm with herniation of parts of small and large intestine and left kidney into the left thoracic cavity. Intestinal coils and kidney were mobilized from thoracic cavity to abdomen without difficulty. Then the rent was repaired with single layer nonabsorbable suture. His postoperative recovery was uneventful and he was keeping well on followup.

## 3. Discussion

The traumatic DH was apparently reported by Sennertus in 1541 [4]. Ambroise Pare described the first case of DH at autopsy in 1579. Antemortem diagnosis of traumatic DH was first made by Bowditch in 1853 and the first successful repair was done by Riolfi in 1886 [5].

The reported incidence of diaphragmatic rupture is between 0.8 and 1.6 percent of the patients admitted to the hospital with blunt trauma. The incidence rises upto 15% among patients with penetrating trauma [4]. Male to female ratio of traumatic DH is 4 : 1 and most of them present in the third decade of life.

A 75% of rupture is from blunt abdominothoracic trauma of severe grade (mainly motor vehicle accidents) and 25% from penetrating trauma (from gunshot and stab injury) [6]. Rare causes of rupture include vomiting, coughing, exercise, pregnancy, and iatrogenic injury. Mechanisms of rupture of diaphragm include (a) sudden increase in intrathoracic/intra-abdominal pressure against a fixed diaphragm, (b) shearing stress on a stretched diaphragm, and (c) avulsion of the diaphragm from its point of attachment [7]. Commonest site of rupture is the posterolateral surface along the embryonic fusion line because it is the weakest part of the diaphragm. Left sided rupture occurs in 70%–80% cases, right sided rupture in 15%–24% cases, and bilateral in 5%–8% cases [8]. The greater prevalence of left sided rupture is due to buffer effect of the liver, embryonic weakness of left hemidiaphragm, and underdiagnosis of right diaphragmatic rupture [9–11]. Intrathoracic kidney is very rare and can occur in three possible situations like congenital DH, traumatic DH, or a congenital ectopic kidney. Herniation of left kidney sometimes occurs in Bochdalek hernia which is more common in new born [12].

Grimes has appropriately described the presentation into three phases—the acute phase, latent phase, and obstructive phase [13]. Acute phase is dominated in 95%–100% cases by associated injuries like rib fracture, pelvic fracture, splenic rupture, closed head injury, liver laceration, haemothorax, pneumothorax, and pulmonary contusion. The diagnosis is frequently missed in the acute phase because of the presence of shock, respiratory failure, concomitant visceral injury, and coma which dominate the clinical picture. Delayed presentation of the DH in the latent phase is with upper gastrointestinal symptoms, chest pain, and dyspnea or an abnormal chest radiograph without symptoms. Patients with obstructive phase often present months to years later with incarceration, obstruction, strangulation, or perforation.

Delayed presentation may be due to delayed detection or delayed rupture [14], the former being more likely. Detection may be delayed if diaphragmatic tear remains asymptomatic at the time of injury and manifests only when hernia occurs. Delayed rupture is possible if diaphragmatic tissue is devitalized at the time of injury but maintains a tenuous barrier until several days later when superadded inflammation weakens it.

Diagnosis of traumatic DH is done preoperatively in 43.5% cases, incidentally at laparotomy or thoracotomy or autopsy in 41.3% cases, and a delayed diagnosis in 14.6% cases [6]. Although chest radiograph is the initial diagnostic modality [15], it is diagnostic only in 33% cases of left sided rupture and 18% cases of right sided rupture [16]. Pathognomonic signs include visualization of herniated stomach or bowel in the chest and extension of intragastric tube above the level of diaphragm [16–18]. Suggestive signs are irregularity of the diaphragmatic contour, elevated diaphragm in the absence of atelectasis, contralateral mediastinal shift in the absence of pulmonary or pleural cause, and persistent basal opacity mimicking collapse or supra-phrenic mass [16–18]. Immediate intubation or presence of concomitant pleural effusion or haemothorax may reduce the diagnostic accuracy of chest X-ray. CT scan of the thorax is a useful aid in the diagnosis in urgent or uncertain cases. The important signs are sharp discontinuation of the diaphragm, intrathoracic visceral herniation, lack of visualization of the diaphragm (absent diaphragm sign), and constriction of bowel or stomach at the site of herniation (collar sign) [19–22]. Thoracoscopy in the acute phase within 24 hours of injury or laparoscopy in delayed cases is advised as alternative diagnostic tool [6].

In the acute presentation, operation is mandatory as soon as proper resuscitation is made. Laparotomy is advocated in this stage to look for concomitant abdominal injury, though some advocates thoracotomy in acute DR of right side for better access and repair [23]. Thoracotomy (if required, extended into a thoracoabdominal incision) is advised in the patients with delayed presentation since the adhesions within the chest can be freed easily and reduction and repair of hernia will be easily accomplished. Laparoscopy and video-assisted thorascopic surgery are newer options in patients with delayed presentations as they require hemodynamically stable state. Preoperative examination of the border of the rent in the diaphragm will help in the differentiation between traumatic and congenital DH. If the border is smooth, it is likely a congenital hernia whereas irregular border suggests traumatic rupture. The latter was the finding in this case.

In conclusion, traumatic DH is often missed if it occurs after seemingly innocuous injury or has a delayed presentation. Diagnosis should be reached as early as possible to reduce mortality. To make radiological diagnosis, radiologists should be familiar with a variety of imaging presentation of injury and maintain a high index of suspicion. In a clinically suspected case, if the chest X-ray is noncontributory, further imaging should be advised. Open surgery or endoscopic surgery should be performed immediately to diagnose and treat the injury.

## References

[1] Hegarty MM, Bryer JV, Angorn IB, Baker LW (1978). Delayed presentation of traumatic diaphragmatic hernia. *Annals of Surgery*.

[2] Carter BN, Giuseffi J, Felson B (1951). Traumatic diaphragmatic hernia. *The American Journal of Roentgenology, Radium Therapy, and Nuclear Medicine*.

[3] Cohen Z, Gabriel A, Mizrachi S, Kapuler V, Mares AJ (2000). Traumatic avulsion of kidney into the chest through a ruptured diaphragm in a boy. *Pediatric Emergency Care*.

[4] Bosanquet D, Farboud A, Luckraz H (2009). A review diaphragmatic injury. *Respiratory Medicine CME*.

[5] Rekha A, Vikram A (2010). Traumatic diaphragmatic hernia. *Sri Ramachandra Journal of Medicine*.

[6] Shah R, Sabanathan S, Mearns AJ, Choudhury AK (1995). Traumatic rupture of diaphragm. *Annals of Thoracic Surgery*.

[7] Cameron JL (2001). Diaphragmatic injury. *Current Surgical Therapy*.

[8] Eren S, Kantarci M, Okur A (2006). Imaging of diaphragmatic rupture after trauma. *Clinical Radiology*.

[9] Ala-Kulju K, Verkkala K, Ketonen P, Harjola P-T (1986). Traumatic rupture of the right hemidiaphragm. *Scandinavian Journal of Thoracic and Cardiovascular Surgery*.

[10] Boulanger BR, Milzman DP, Rosati C, Rodriguez A (1993). A comparison of right and left blunt traumatic diaphragmatic rupture. *Journal of Trauma*.

[11] Andrus CH, Morton JH (1970). Rupture of the diaphragm after blunt trauma. *The American Journal of Surgery*.

[12] Obatake M, Nakata T, Nomura M (2006). Congenital intrathoracic kidney with right Bochdalek defect. *Pediatric Surgery International*.

[13] Grimes OF (1974). Traumatic injuries of the diaphragm. Diaphragmatic hernia. *The American Journal of Surgery*.

[14] Johnson CD (1988). Blunt injuries of the diaphragm. *British Journal of Surgery*.

[15] Goh BKP, Wong ASY, Tay K-H, Hoe MNY (2004). Delayed presentation of a patient with a ruptured diaphragm complicated by gastric incarceration and perforation after apparently minor blunt trauma. *Canadian Journal of Emergency Medicine*.

[16] Gelman R, Mirvis SE, Gens D (1991). Diaphragmatic rupture due to blunt trauma: sensitivity of plain chest radiographs. *American Journal of Roentgenology*.

[17] van Vugt AB, Schoots FJ (1989). Acute diaphragmatic rupture due to blunt trauma: a retrospective analysis. *Journal of Trauma*.

[18] Groskin SA (1992). Selected topics in chest trauma. *Radiology*.

[19] Kang E-Y, Müller NL (1996). CT in blunt chest trauma: pulmonary, tracheobronchial, and diaphragmatic injuries. *Seminars in Ultrasound CT and MRI*.

[20] van Hise ML, Primack SL, Israel RS, Müller NL (1998). CT in blunt chest trauma: indications and limitations. *Radiographics*.

[21] Worthy SA, Kang EY, Hartman TE, Kwong JS, Mayo JR, Müller NL (1995). Diaphragmatic rupture: CT findings in 11 patients. *Radiology*.

[22] Murray JG, Caoili E, Gruden JF, Evans SJJ, Halvorsen RA, Mackersie RC (1996). Acute rupture of the diaphragm due to blunt trauma: diagnostic sensitivity and specificity of CT. *American Journal of Roentgenology*.

[23] Kozak O, Mentes O, Harlak A (2008). Late presentation of blunt right diaphragmatic rupture (hepatic hernia). *American Journal of Emergency Medicine*.

